# A curated image dataset for sapodilla fruit (Manilkara zapota) disease and fruit quality analysis

**DOI:** 10.1016/j.dib.2026.113004

**Published:** 2026-06-21

**Authors:** Jameer kotwal, Atharva Athanikar, Shubham Kumbhar, Devdatt Khilari, Sahil Borkar

**Affiliations:** a Dr. D. Y. Patil Institute of Technology, Pune, 411018, India; b Dnyaan Prasad Global University, School of Technology and Research, Pune, 411018, India

**Keywords:** Sapodilla fruit (Manilkara zapota), Datasets, Image classification, Deep learning

## Abstract

Sapodilla (Manilkara zapota), or chickoo, is a key tropical fruit, very popular in India, Mexico, and Thailand, as it is nutritionally and economically valuable. Nonetheless, the production of sapodillas is often affected by several diseases, which reduce fruit quality and quantity. The dataset used in this paper is a sapodilla fruit image dataset, comprising 1,518 images, gathered in the field under the practicing conditions on 18 February 2025, 22 February 2025, in Rahu village, Pune district, Maharashtra, India, with the use of smartphone cameras. The data is sorted into four categories, namely: Anthracnose, Bacterial rot, Healthy, and Sap bleeding. The photographs were taken in different backgrounds and in different lighting conditions to represent real-life cultivation conditions. The data is expected to be useful in machine learning-based plant disease detection, classification, and analysis, and spur the creation of intelligent and sustainable agricultural systems.

Specifications Table

**Table:** 

Subject	Computer Sciences
Specific subject area	Sapodilla fruit (Manilkara zapota), computer vision
Type of data	Raw field-acquired images of sapodilla fruits
Data collection	Smart phone: Vivo V27, focal length: 6 mm
Data source location	Locality Name: Rahu (राहू), Taluka Name: Daund, District: Pune State: Maharashtra, India-412207, latitude: 18.5711° N, longitude: 74.2705° E
Data accessibility	Repository name: Sapodilla (Manilkara zapota) Fruit: A Comprehensive Approach for Agricultural Automation Data identification number: Version: V1, Doi: 10.17632/xbzd2fjd3p.1 Direct URL to data: https://data.mendeley.com/datasets/xbzd2fjd3p/1 Website Designed (Link): https://kisanai.site/
Related research article	**Case study:** **Article Title:** Detection of Sapodilla Fruit Diseases Using EfficientNet V2 Large: A Comprehensive Approach for Agricultural Automation **Author:** Jameer kotwal, Atharva Athanikar, Shubham Kumbhar, Devdatt Khilari, Sahil Borkar **Paper Link:**https://ieeexplore.ieee.org/document/11306488 [1] **Conference name:** IEEE International Conference on Electronics, Computing and Communication Technologies (CONECCT) **Publisher:** IEEE

## 1 Value of the Data

• The data offers a labeled collection of images of sapodilla (Manilkara zapota) fruits of four classes: Anthracnose, Bacterial rot, Healthy, and Sap bleeding.
• The information is applicable in machine learning and deep learning-related detection and classification of plant diseases in the real fields [2].
• The dataset can be used to train, test, and benchmark models in the field of researchers dealing with computer vision, precision agriculture, and smart farming [3].
• The dataset helps in developing early disease diagnosis systems to enhance the management of crops and sustainable agricultural practices [4].
• The dataset can also be used to enable transfer learning applications, such as fine-tuning models trained on the sapodilla fruit diseases to detect disease in other tropical fruit crops, which may have limited labelled data.

## 2 Background

Chickoo, also called sapodilla (Manilkara zapota), is a highly important tropical fruit with a valuable economic significance in such areas as India, Mexico, and Thailand. Due to the sweetness and nutritional value, the fruit is treasured, but the levels of sapodilla production are often influenced by diseases, which negatively affect the quality of the fruit and its price in the market. Conventional methods of disease detection are dependent on a manual examination by either the grower or an expert and thus time-consuming and subjective, especially in extensive cultivation fields.

The promotion of image-based datasets used to detect automated plant disease has been promoted by recent progress in computer vision and machine learning. Although some open data sets are on major crops, there are few annotated image datasets on sapodilla fruit diseases, especially on actual fields. This is a deficiency in data that limits the creation and testing of a computational model of sapodilla health assessment [5].

## 3 Data Description

The dataset includes 1,518 digital images of sapodilla (Manilkara zapota) fruits collected in field conditions. All picture files are organized into hierarchical folders by the fruits' health conditions. The dataset root folder contains four main folders, each associated with a particular class label at https://data.mendeley.com/datasets/xbzd2fjd3p/1. The folder Anthracnose has pictures of the sapodilla fruits with noticeable symptoms of anthracnose infection. The sub-folder of bacterial rot contains photographs of fruits with bacterial rot. Images of sapodilla fruits that are healthy for the eyes and show no sign of the disease are placed in the Healthy folder. Sap bleeding: a folder contains photos of the symptoms of sap exudation on the surface of the fruits [6,7].

**Fig. 1:**
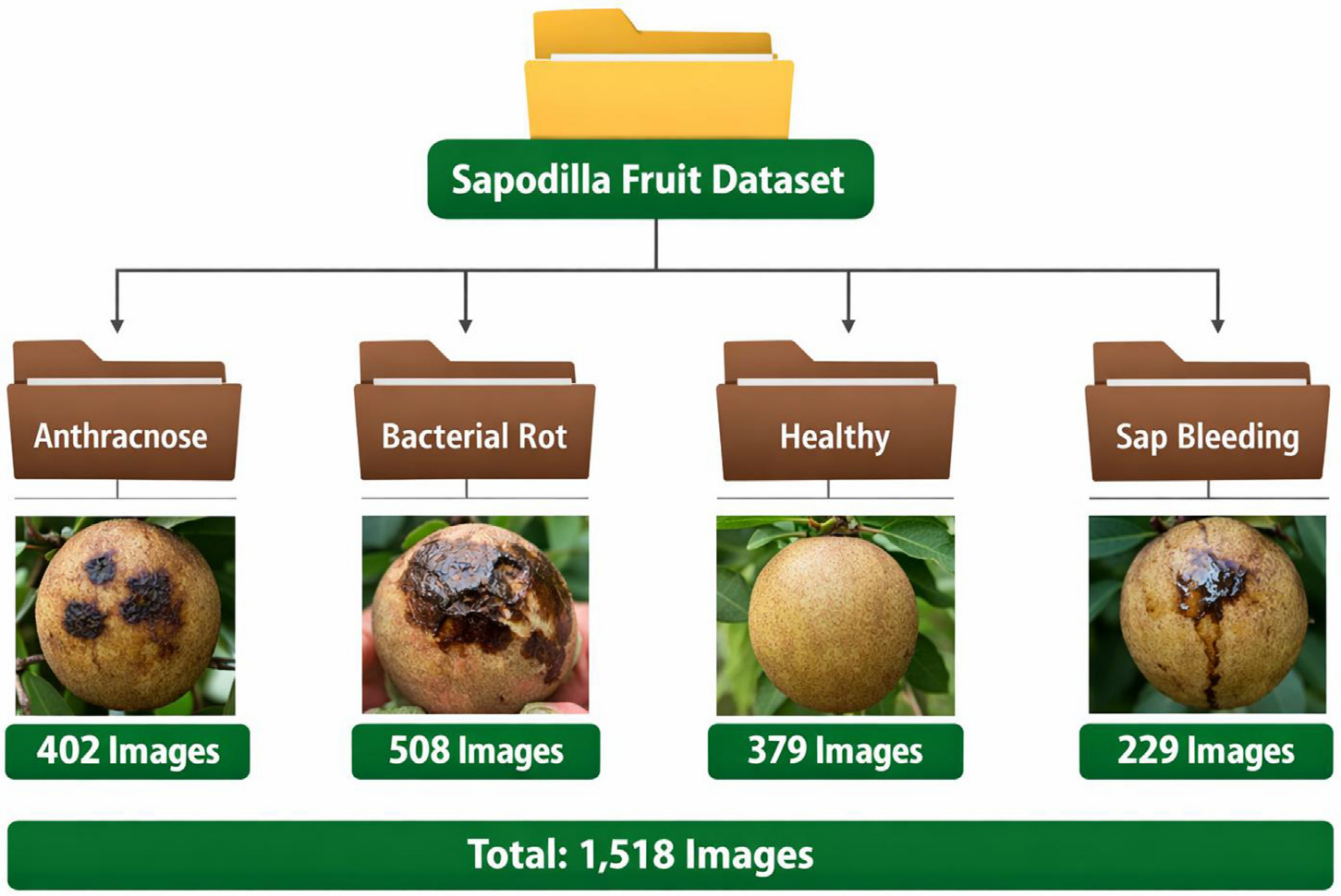
Sapodilla fruit dataset folder and number of images.

The smartphone cameras were used to take all the images, and the image files were stored in the usual image file formats. The dataset occupies approximately 8.14 GB of storage space on Mendeley Data. The average image resolution is approximately 4096 × 3072 pixels, with 72 × 72 dpi and bit depth 24, with slight variations depending on the smartphone device and camera settings. Photographs were taken at different angles, distances, and backgrounds. The dataset exhibits moderate class imbalance, which reflects natural disease occurrence under field conditions. The images were not preprocessed, resized, or augmented before storage [8]. The image files are put in one class-specific folder, and there are no duplicate images across folders, as shown in Fig. 1. Table 1 shows the number of images and types of diseases.

**Table 1:** Number of images with the type of disease.

Category	Description	Number of images
Anthracnose	Images of sapodilla fruits affected by anthracnose disease	402
Bacterial rot	Images of sapodilla fruits affected by bacterial rot	508
Healthy	Images of healthy sapodilla fruits	379
Sap bleeding	Images of sapodilla fruits showing sap bleeding symptoms	229
**Total**		**1,518**

The dataset was designed considering plant characteristics, orchard conditions, and image acquisition parameters. The following are some details:

1) Plant & Orchard Details

a) What sapodilla variety/cultivar was used?
b) Were all plants from the same orchard/location?
c) Were plants naturally grown or managed?

Answer: The sapodilla fruits were collected from locally cultivated sapodilla trees grown in a single agricultural location in Rahu village, Pune district, Maharashtra, India. The specific cultivar/variety was not recorded at the time of data collection. All plants were grown naturally and managed conventionally using common agricultural practices followed by local farmers. The sapodilla plants were mature, fruit-bearing trees, with an approximate age range of 6 to 10 years.

2) Disease Labeling Details

a) How were disease classes identified?
b) Were multiple observers involved in labeling?
c) Were images with ambiguous symptoms excluded?

Answer: Disease categories were assigned based on visible symptoms observed on the fruit surface. Labeling was carried out through manual visual inspection at the time of data collection. Consultation with experienced agricultural practitioners and farmers who are knowledgeable and used to the symptoms of sapodilla disease was undertaken during the labeling process. Ambiguous and overlapping images were omitted to assure consistency of labels. Fruits showing clear and distinguishable symptoms were included in the dataset. Images with ambiguous, unclear, or overlapping symptoms were excluded to maintain label consistency. Each image was assigned to one class label only, and no image belongs to more than one category.

3) Image Properties

a) What is the image resolution range?
b) What file format was used?
c) Were images captured in portrait or landscape mode?

Answer: All images were captured using smartphone cameras. Images are stored in JPEG format. The image resolution varies depending on the smartphone device and camera settings used during capture. Images were captured in both portrait and landscape orientations.

4) Environmental Conditions

a) Approximate time of day of image capture?
b) Were images taken in dry or humid conditions?
c) Was data collection done during a specific season?

Answer: Image acquisition was carried out under natural daylight conditions. Images were captured at different times of the day. Data collection occurred during the local harvesting season in February. Environmental conditions such as temperature and humidity were not artificially controlled or recorded.

5) Dataset Organization

a) Are image filenames unique and anonymized?
b) Is there one label per image?
c) Are there no duplicate images across classes?

Answer: Each image file has a unique filename. Images are organized into four class-specific folders: Anthracnose, Bacterial rot, Healthy, and Sap bleeding. No duplicate images are present across folders. No preprocessing, augmentation, or modification was applied to the images before storage.

## 4 Experimental Design, Materials, and Methods

The dataset was meant to record a visual observation of the sapodilla (Manilkara zapota) fruits in healthy and diseased states. The data collection was done in Rahu village, Pune district, Maharashtra, India, during a predetermined collection time of 18 February 2025 to 22 February 2025.

The smartphone cameras were used to get images with the default settings on the cameras. Photographs were taken during natural daylight setting without artificial light and external photography equipment. The photos of fruits were taken on the plant and after harvest, which guaranteed the reflection of the naturally occurring conditions. The photographs were taken at different distances and positions, with frontal, side, and angled positions, and with different natural backgrounds (foliage, soil, and sky). There was no image enhancement, filtering, resizing, or augmentation in the acquisition of data. All the obtained images were examined manually to exclude the blurred, duplicated, or inappropriately framed samples. The other images were grouped into four classes depending on the overt fruit conditions: Anthracnose, Bacterial rot, Healthy, and Sap bleeding. Labeling was carried out visually in terms of surface features that could be seen.

The last dataset has 1,518 pictures, which are separated into directories with different classes. Images are saved in the standard format of digital images and organized into a hierarchy of folders that can be used in machine learning and computer vision processes. No preprocessing pipelines were used or software tools that were involved in data generation or organization. Table 2 lists the parameters associated with smartphone-based image acquisition.

**Table 2:** Image acquisition parameters.

Parameter	Description
Imaging device	Commercial smartphone cameras
Camera settings	Default settings
Lighting conditions	Natural daylight
Image capture location	On-tree and post-harvest fruits
Viewing angles	Multiple angles (front, side, oblique)
Backgrounds	Natural backgrounds (leaves, soil, sky)
Preprocessing	None applied
Image format	Standard digital image formats

**Fig. 2:**
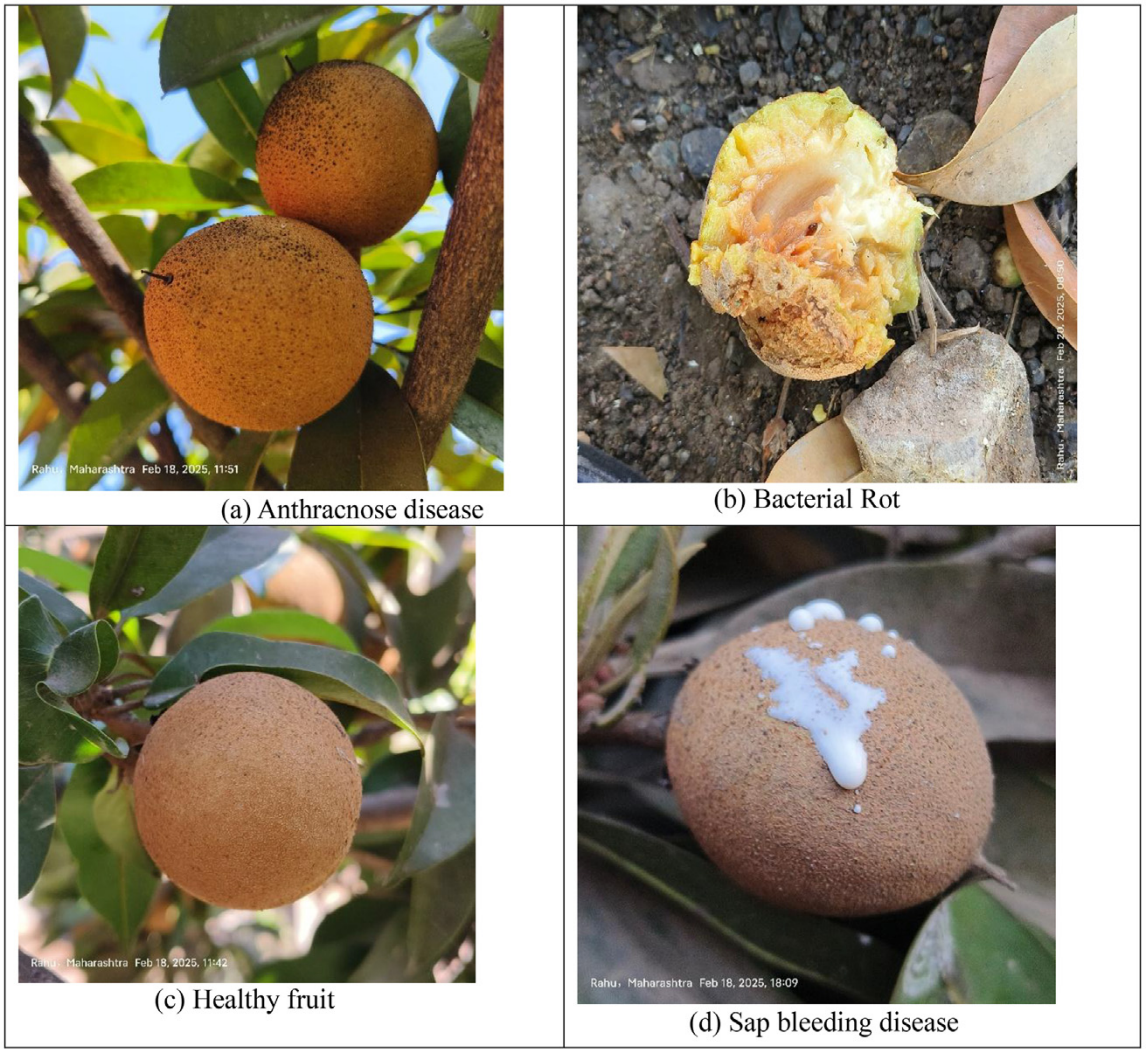
Samples of the four dataset classes: (a) Anthracnose, (b) Bacterial Rot, (c) Healthy, and (d) Sap Bleeding.

Colletotrichum species are the primary causes of anthracnose in sapodilla and result in sunken lesions of the leaf, flower, and fruit, which are dark. It lowers the quality and productivity of fruits, particularly in warm and humid climates, as shown in Fig. 2(a). Sapodilla infected with fungi presents symptoms of dark spots, leaf blight, rotting of fruits, and early dropping of fruits, which are usually caused by such pathogens as Colletotrichum and Phytophthora, as shown in the Figure 2(b). These infections are most prevalent in moist environments, and they produce a substantial impact on the quality and production of fruits. Fig. 2(c) shows the healthy sapodilla fruit. Cases of sapodilla with sap bleeding disease have persistent oozing of milky latex through cracks in the bark, which is usually caused by fungi or mechanical damage, as shown in Fig. 2(d). The illness makes the tree weak and limited in the number of fruits that can be produced and may later cause the destruction of the branch or the plant in case of no control.

## Limitations

The limitations of the dataset are:

• Sapodilla cultivation regional differences were not well represented since the dataset was gathered in only one geographic area (Rahu village, Pune district, Maharashtra, India).
• The sapodilla cultivar or variety was not noted during the collection of data.
• Labeling of the diseases was carried out by the visual observation of the symptoms on the surface of the fruits without laboratory or pathological verification.
• The smartphone cameras were used to take pictures in natural light conditions, and thus there were differences in the light, resolution, and background.
• The data only consists of fruit pictures and does not feature pictures of leaves, stems, and entire plants.
• The conditions of the environment, including temperature, humidity, and the soil, were not tested or recorded when capturing an image.

## Future Work

More diverse and robust datasets will be acquired in future versions by incorporating multi-location acquisition, laboratory-confirmed disease annotations, environmental metadata, and other plant components, including leaves and stems.

## Ethics Statement

• This research presents an accurate account of the work performed, all data presented are accurate and methodologies detailed enough to permit others to replicate the work.
• This manuscript represents entirely original works and or if work and/or words of others have been used, that this has been appropriately cited or quoted and permission has been obtained where necessary.
• This material has not been published in whole or in part elsewhere.
• We haven’t conducted any experiments on humans and animals.

## CRediT Author Statement

**Jameer Kotwal:** Investigation, Software, Writing – original draft, first draft, Writing: review and editing. **Atharv Athanikar:** Resources, Conceptualization, Validation, Software, Data Curation. **Shubham Khumbhar:** Resources, Conceptualization, Validation, Software, Data Curation. **Devdatt Khilari**: Supervision, Data Curation. **Sahil Borkar:** Methodology, Investigation.

## Data Availability

Mendeley DataSapodilla(Manilkara zapota) Fruit: A Comprehensive Approach for Agricultural Automation (Original data).
